# Development of layer-by-layer assembled polyamide-imide membranes for oil sands produced water treatment

**DOI:** 10.1038/s41598-021-87601-4

**Published:** 2021-04-14

**Authors:** Nusrat Helali, Laleh Shamaei, Masoud Rastgar, Mohtada Sadrzadeh

**Affiliations:** Department of Mechanical Engineering, 10-367 Donadeo Innovation Center for Engineering, Advanced Water Research Lab (AWRL), University of Alberta, Edmonton, AB T6G 1H9 Canada

**Keywords:** Environmental sciences, Engineering, Materials science

## Abstract

The sustainable expansion of steam-assisted gravity drainage, as one of the most popular enhanced oil recovery methods, strongly depends on the proper management of the produced water. The strict environmental regulations have forced the oil sands industry to treat and reuse the produced water for oil extraction. Membrane separation as a single-step water treatment technique has played an important role in removing multiple-sized contaminants from wastewater. However, fouling limits the widespread application of this technology if the membrane is not modified properly to achieve antifouling propensities. Herein, we used the layer-by-layer assembly technique to sequentially coat the hydrophilic poly(diallyl dimethylammonium chloride) and polyacrylic acid on the surface of the polyamide-imide porous membrane to improve its fouling resistance. The effect of the number of bilayers on fouling and permeation properties was examined. The membrane with the highest fouling resistance and reasonable hydrodynamic permeability of 5.2 LMH/psi was achieved by coating four bilayers. This membrane exhibited a low flux decline of 50.2% and a high flux recovery ratio of 100%, while these numbers for the pristine PAI membrane were 75.9% and 97.8% under similar test conditions. The enhanced antifouling characteristics of the modified membranes indicate the viability of these membranes for oil sands produced water treatment with an easy cleaning procedure. The key parameter that contributed to the enhanced fouling resistance of the bilayer-coated membranes was the improved surface hydrophilicity, which manifests through the reduction of water contact angle from 62° ± 3° for the pristine membrane to 52° ± 2° for surface-modified membranes.

## Introduction

Over the past decades, continuous population growth, industrial development, and lack of proper wastewater management have led to the increasing freshwater demand. The consequent global shortage of unimpaired freshwater has imposed the necessity for efficient wastewater treatment for recycling or safe disposal to the environment. Canada's oil sands industry is one of the largest suppliers of world energy whose sustainable expansion depends strongly on the treatment and reuse of the produced water during resource extraction^1^. Steam-assisted gravity drainage (SAGD) is a thermally enhanced heavy oil recovery method for producing bitumen that is widely adopted by oil sands industries in Alberta, Canada^2–4^. During this process, a large amount of water is used to produce hot steam being injected into bitumen formation through a horizontal injection well to reduce the viscosity of bitumen^5, 6^. Afterward, the mixture of bitumen and condensed vapor is pumped up to the surface through the production well, and bitumen is separated from the water. Finally, the produced water is treated, recycled, and reused as the steam generator feed, also known as boiler feed water (BFW)^7^. SAGD operation uses roughly 0.6 to 0.9 barrels of freshwater to extract one barrel of bitumen^8, 9^. Therefore, environmental and economic concerns regarding the consumption of such huge fresh water in the oil sands industry have forced efforts toward developing more efficient techniques for the treatment of SAGD produced water.

Membrane technology has revolutionized the separation industry due to its advantages, including high water quality, low footprint, and compact modular structure^3, 10–12^. However, large-scale deployment of membranes in the oil sands industry has been restricted by concerns regarding the severe fouling of membranes^13, 14^. Fouling is caused due to the accumulation of contaminants on the membrane surface and/or inside the pores and can hinder the permeation through the membranes and increase the operating cost of the treatment process^15, 16^. Improving the surface hydrophilicity of membranes has been widely investigated as an indispensable technique to enhance the fouling resistance^17–20^. The hydrophilic functional groups on the membrane surface can adsorb water molecules and form a hydration layer that can repel the foulants^15^.

Among various materials that have been employed as the host polymer for the fabrication of membranes, polyamide-imide (PAI), commercially known as Torlon, has shown superior mechanical and chemical stability over a wide range of pH^21^. Despite the promising properties of PAI membranes, only a few works studied the effect of the surface modification techniques on the antifouling properties permeselectivity of PAI membranes. Zhang et al.^22^ crosslinked the surface of PAI hollow fiber substrates with hyperbranched polyethyleneimine (PEI), graphene oxide (GO) sheets, and ethylenediamine. The fabricated membrane showed great potential for the removal of heavy metals (> 95% for Pb, Ni, and Zn) without sacrificing the water permeability^22^. Goh et al.^23^ also used PEI as the crosslinking agent, followed by GO coating. The GO-modified hollow fiber PAI membranes provided higher selectivity for the separation of CaCl_2_ than unmodified membranes (> 83% rejection as compared to ~ 70%) and 86% higher pure water^23^. Also, the PEI crosslinking time was found to decrease the water flux significantly. Setiawan et al.^24^ crosslinked PEI to the surface of PAI microporous hollow fiber substrates and tested the modified membranes in ultrafiltration (UF) and forward osmosis (FO) processes. In UF tests, the MgCl_2_ rejection increased from 11.9 for the pristine membrane to 94.4% for the PEI-crosslinked membrane at the expense of a significant decline in pure water flux from 26.7 LMH to 1.8 LMH at 1 bar pressure. Using 0.5 M MgCl_2_ as the draw solution in FO tests, the optimum crosslinked membrane provided 9.7 LMH water flux and 0.4 g L^−1^ reverse solute flux to water flux ratio, which was reported to be 100% better than the studied commercial membranes^24^. Sun et al.^21^ crosslinked the surface of the PAI membrane with hyperbranched PEI with different molecular weights to fabricate positively charged nanofiltration (NF) hollow fiber membranes. They observed that PEI modification dramatically increased the rejection of MgSO_4_, NaCl, and MgCl_2_ to more than 95% for the highest molecular weight PEI. Also, increasing the molecular weight of PEI from 2 to 60 kDa increased the salt rejection by almost 40%, which was attributed to the decrease in the effective pore radius of the membranes after PEI crosslinking (from 0.46 nm to 0.40 nm)^21^. Li et al.^25^ used allylamine hydrochloride and glutaraldehyde as the crosslinking agents to fabricate UF and PRO hollow fiber membranes. It was observed that the optimized membrane removed more than 80% of different organic solutes (glycerol, ribose, and glucose) and salts (MgSO_4_, MgCl_2_, and Na_2_SO_4_) in the UF process and provided stable power output of 4.3 W m^−2^ at ~ 12 bar hydraulic pressure in PRO^25^. Ong et al.^26^ functionalized the surface of PAI hollow fiber membranes with PEI to synthesize NF membranes with more than 90% rejection against different dyes (reactive blue 19, reactive black 5, and reactive yellow 81) and a high antifouling tendency (~ 20% flux decline and ~ 100 flux recovery after chemical cleaning). Shakeri et al.^27^ employed a facile pressure-assisted technique to coat the surface of PAI membranes with polyaniline and dimethyl sulphoxide. The fabricated electro-conductive membranes were tested in the FO process and showed remarkable fouling resistance against sodium alginate. By applying 2 V electric potential, both flux recovery and flux decline rates improved by more than 20% and 35%, respectively. Wang et al.^28^ crosslinked PEI on the surface of the flat sheet PAI substrate to change the surface charge of the membrane from negative to positive, making them suitable for colored wastewater treatment. The crosslinked membranes exhibited high rejection (> 95%) and antifouling properties (> 90% flux recovery rate and < 25% flux decline rate) against basic dyes with different molecular weights (320–1299 g mol^−1^).

Despite promising results, all studies mentioned above suffer from one major drawback. The high crosslink density of the coating layer, primarily originating from PEI in most of these studies, has led to the fabrication of dense NF and FO membranes. This challenge can be mitigated by employing the versatile and straightforward layer-by-layer (LbL) assembly technique, where an ultrathin multilayer with controllable thickness, surface potential, permeability, and structure can be coated to enhance the fouling resistance of membranes^29–31^. LbL-assembly is driven by molecular interactions, such as electrostatic forces, hydrogen bonding, interatomic charge-transfer linkage, covalent bonding, stereo-complexation, and surface sol–gel process^29^. The basic idea is to alternately deposit anionic and cationic polyelectrolytes (PEs) on the membrane's surface^32–34^.

In this work, the LBL assembly technique was carried out to fabricate cost-effective UF membranes with improved antifouling properties. To the best of our knowledge, this is the first work that studied the application of the LBL-assembly technique for the fabrication of flat-sheet PAI UF membranes. Polydiallyldimethylammonium chloride (pDAC) and polyacrylic acid (PAA) were used as the polycation and the polyanion, respectively. The effect of the number of the PEs alternative bilayers was examined to obtain a desirable permeation and antifouling performance for the modified membranes. The antifouling properties of the modified membranes were investigated by filtration of SAGD BFW. The change in the separation efficiency and the antifouling properties of the fabricated membranes were justified by the study of their surface morphology, charge, and wettability. The use of PAI with abundant polarizable imide and amide functional groups was found to facilitate the formation of a robust and closely packed LbL-assembled multilayer.

## Materials and methods

### Chemicals and reagents

**Figure 1:**
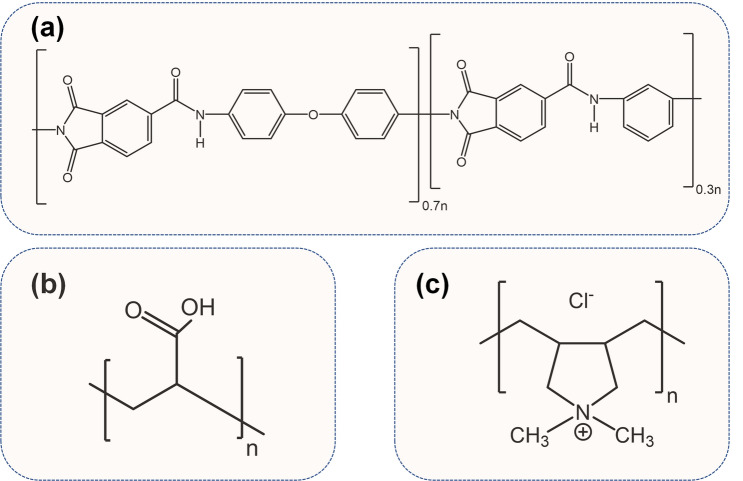
Molecular structures of (**a**) polyamide-imide (PAI), (**b**) polyacrylic acid (PAA) as the polyanion, and (**c**) polydiallyldimethylammonium chloride (pDAC) as the polycation^35–37^.

PAI (Torlon 4000 T-HV, Solvay Advanced Polymers, GA, United States) as the main polymer, polyvinylpyrrolidone (PVP, reagent grade Mw. 10 kDa) as an additive, and N, N-dimethylacetamide (DMAc, 99%, Sigma Aldrich) as a solvent were used to fabricate the support membrane for the LbL deposition. PAA (Mw. 450 kDa) and pDAC (Mw. 200–350 kDa, 20 wt % in water solution) were purchased from Sigma Aldrich and utilized as polyanion and polycation, respectively. The molecular structures of PAI, pDAC, and PAA are presented^35–37^ in Fig. 1. Deionized (DI) water (18.2 MΩ cm^−1^, Milli-Q, Millipore) was used to prepare polyelectrolytes aqueous solutions. SAGD produced water, provided from a bitumen extraction plant in the Athabasca oil sands region, Alberta, Canada, was used as industrial wastewater to conduct the antifouling tests. The employed SAGD produced water is the boiler feed water (BFW), which is the inlet of steam generators. Samples are collected, shipped in sealed containers, and were kept in an inert atmosphere (under nitrogen blanket). The specification summary of the BFW is presented in Table 1. The pH of BFW was measured by a Mettler Toledo (SevenMulti S47) pH meter. Inductively coupled plasma-optical emission spectroscopy (ICP-OES, Agilent 735) was used to measure the concentration of inorganic materials in the BFW.

**Table 1:** The characteristics of SAGD BFW at 25 °C.

Parameter	Units	Specification
Conductivity	mS/cm	2.0
TDS	mg/L	2000
pH	–	10.5
TOC	mg/L	500
Dissolved silica	mg/L	30
Ca^2+^	mg/L	0.5
Iron	mg/L	0.3
Zeta potential	mV	− 34

The BFW was used as the feed solution to evaluate the antifouling properties of the fabricated membranes.

### Evaluation of the surface potential of the PE solutions

The surface charge of the polyelectrolyte particles was evaluated using a Zetasizer Nano ZS90 (Malvern Instruments Ltd., UK) with a 633 nm red laser and a folded capillary cell (DTS1060). This device measures zeta potential using Laser Doppler Micro-electrophoresis. The suspensions of the polyelectrolyte particles were made on the same day and immediately before the analysis. DI water was used as the solvent.

### Preparation of the LbL-assembled membranes

Asymmetric PAI membranes with the molecular weight cut-off of 700 kDa were synthesized via a nonsolvent-induced phase separation technique. In the first step, 14 wt% of PAI and 2 wt% PVP were dissolved in DMAc, and the mixture stirred at 100 rpm overnight. Afterward, the mixture was allowed to settle for 10 h for the complete release of air bubbles. The cast solution was then poured over a polyester fabric support and a micrometer film applicator (Gardco, Pompano Beach, FL, USA) with a clearance gap of 200 μm was employed to spread the cast solution over the fabric support. An automatic film applicator (TQC Sheen, AB3120, The Netherlands) was used to perform the casting process at a constant speed of 10 mm s^−1^. The cast film was then immersed into a bath of distilled water for 15 min. Finally, the fabricated membrane was soaked into distilled water overnight to remove the residual solvent.

For the LBL assembly, the PE solutions with a concentration of 0.01 wt% were first prepared by dissolving cationic pDAC and anionic PAA in DI water. The PAI substrate (M0) was then placed between rubber gaskets and acrylic frames with the active surface facing up, and 20 ml of 0.01 wt% pDAC solution was poured over the substrate and left for 5 min. Next, the excess pDAC solution was discarded and the membrane was submerged in DI water for 20 min to wash the loosely attached polyelectrolytes. After that, the same amount of anionic PAA solution was deposited on the pDAC-coated membrane and allowed to stay for 5 min, followed by washing to form the first bilayer. Similar steps were repeated sequentially to form more bilayers. To study the effect of the number of bilayers on the permeation and antifouling performances, three membranes were synthesized by coating the pristine PAI support (M0) with 1, 2, and 4 bilayers of 0.01 wt% PE solutions and named M1, M2, and M3, respectively. The successful deposition of PEs was confirmed by zeta potential measurement.

### Evaluation of the surface potential of the membranes

The surface zeta potentials of the fabricated membranes were measured using a Surpass 3 Electrokinetic analyzer (Anton Paar, Graz, Austria). The zeta potential values were evaluated over the pH range of 3–9 at a temperature of 25 ˚C using a 1 mM KCl solution.

### Evaluation of the chemical compositions of the membranes

The chemical composition of the fabricated membranes was studied using Energy-dispersive X-ray (EDX) spectroscopy and attenuated total reflectance-Fourier transform infrared spectroscopy (ATR-FTIR). An SEM–EDX (Bruker model) with dual silicon drift detectors was employed to perform the EDX analysis. ATR-FTIR spectra were recorded in the air at room temperature employing an Agilent Technologies, Cary 600 series FTIR spectrometer. Thirty scans were recorded for each sample with a resolution of 4 cm^−1^ and over the range of 400–4000 cm^−1^.

### Evaluation of the surface topography of the membranes

Field emission scanning electron microscopy (FESEM, Zeiss Sigma 300 VP) at 10 kV acceleration voltage was used to study the surface morphology of the fabricated membranes. Prior to imaging, all the samples were sputter-coated with a thin carbon film. The surface topography of the synthesized membranes was investigated employing atomic force microscopy (AFM, Bruker Dimension Icon, USA). AFM measurements were conducted in tapping mode under ambient temperature and humidity at a scan rate of 1.0 Hz over a 10 μm × 10 μm surface area of the samples. The analysis of the AFM data and the calculation of the surface roughness parameters were performed using Nanoscope analysis software V.1.40.

### Evaluation of the surface wettability of the membranes

To study the surface wettability of the membranes, measurements of the water in air contact angles were carried out by sessile drop method at ambient conditions using a Krüss DSA 100 (Krüss GmbH, Germany) instrument. A 2 µl droplet of DI water was deposited on the surface of the membranes using a micro-syringe, and the water contact angle was measured after equilibrium was reached. Five measurements were performed at different locations of each sample, and the average contact angles were reported.

### Evaluation of the permeation performance of the membranes

**Figure 2:**
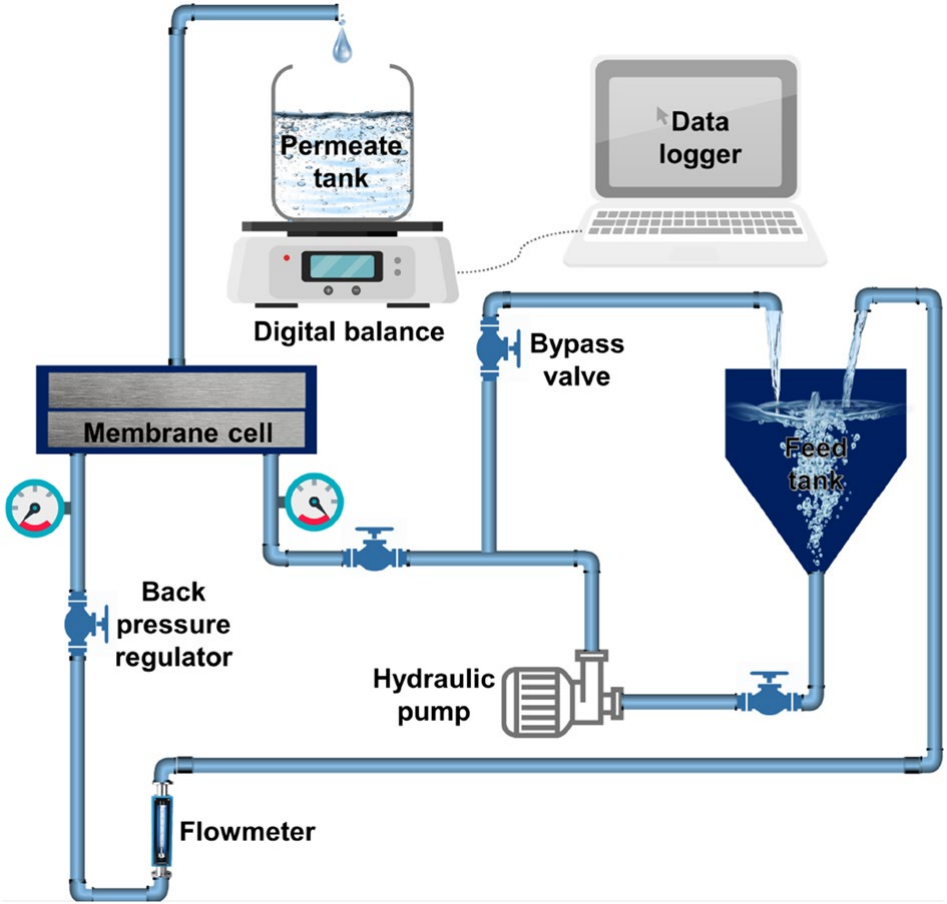
Schematic view of the crossflow filtration setup.

The permeation performance of the fabricated membranes was evaluated using a crossflow membrane filtration setup. Figure 2 shows the process flow diagram of the setup. The setup is equipped with a diaphragm pump (HydraCell M03) to provide the hydraulic transmembrane pressure (TMP). The synthesized membranes were mounted on a module with an effective filtration area of 20.6 cm^2^. Prior to each experiment, the fabricated membranes were compacted at 40 psi until a steady water flux was obtained. Permeation tests were performed at a constant feed flow rate of 1.6 LPM (Liters per minute). A weighing balance (EL4001, Mettler Toledo, USA) was also used to record the mass of permeate at a regular time interval. The pure water flux (*J*_*w*_) is calculated using the equation below^38^:

$$J_{w} = \frac{m}{A\rho \Delta t}$$

where m (kg) is the mass of permeate, A (m) is the effective filtration area, ρ (kg m^−3^) is water density, and Δt (h) is the time difference between each record. A series of filtration tests with distilled water was performed at different TMP, and the hydrodynamic permeability (A) was defined as the slope of the flux-TMP plot.

### Evaluation of the antifouling properties of the membranes

SAGD BFW, as industrial wastewater, was used to perform the fouling tests. Before each test, the membranes were compacted at 40 psi using pure water to reach a steady flux. Afterward, the initial permeate flux (*J*_*wi*_) of all filtration tests was maintained constant at 140 LMH by adjusting the TMP to disregard the effect of the permeation drag on the flux decline of the membranes^39, 40^. The pure water was then replaced with SAGD BFW and the permeation flux (*J*_*f*_) was recorded for 1 h. After that, the surface of the membrane was washed with pure water at atmospheric pressure and the recovered pure water flux (*J*_*wr*_) was recorded. The feed flow rate was maintained at 1.6 L min^−1^ (LPM) in all experiments. Permeate was collected, and the rejection of organic compounds was measured using a UV–Vis spectrophotometer (Thermo Fisher Scientific GENESYS™ 10) at a wavelength of 290 nm. The rejection percentage (R%) of the organic matters, percentage of flux decline (FD), and flux recovery ratio (FRR) are calculated using the following equations^15, 38^:

1 $$R\left( \% \right) = \left[ {1 - \frac{{C_{permeate} }}{{C_{Feed} }}} \right] \times 100$$

2 $$FD = 1 - \frac{{J_{f} }}{{J_{wi} }} \times 100.$$

3 $$FRR = \frac{{ J_{wr} }}{{J_{wi} }} \times 100$$

where *C*_*permeate*_ and *C*_*Feed*_ are the organic matter concentration in the permeate and feed solutions, respectively.

## Results and discussion

### Evaluation of the surface charge of the membranes

**Figure 3:**
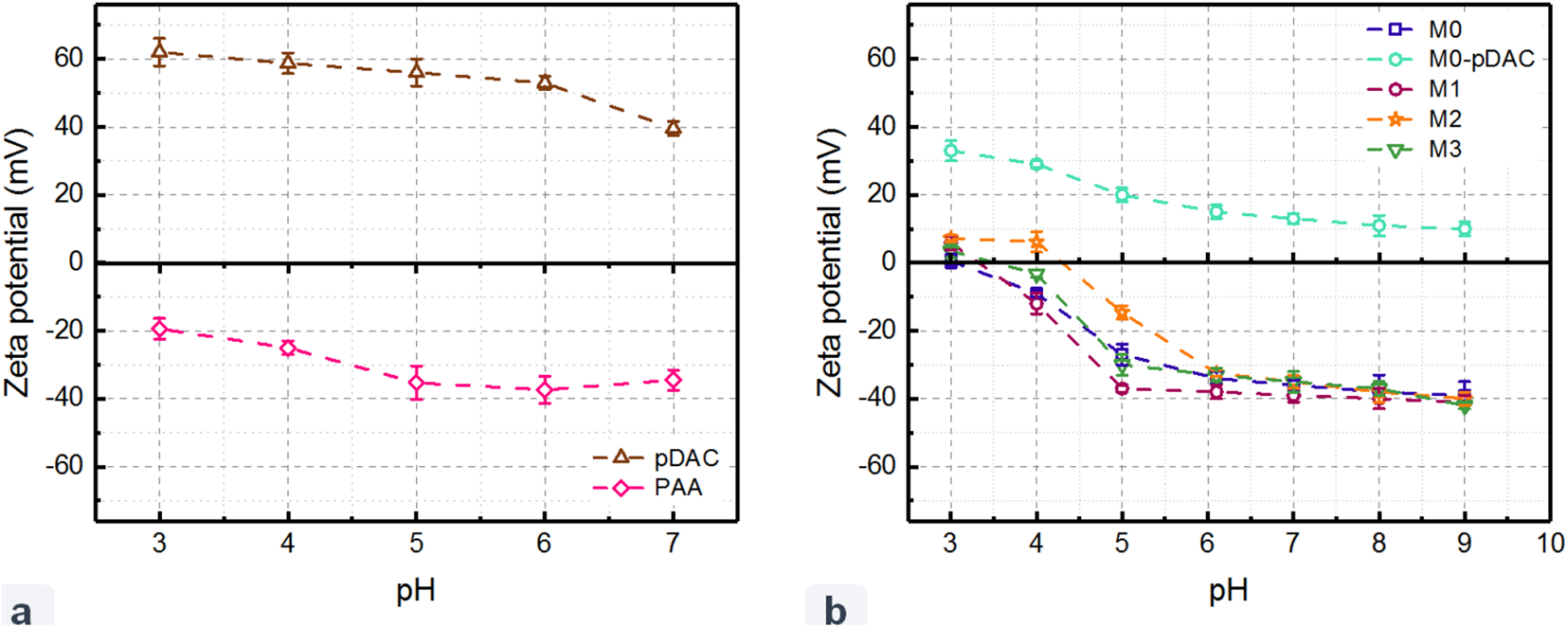
The surface zeta potentials of (**a**) pDAC as polycation and PAA as polyanion, and (**b**) Fabricated membranes. The modified membranes M1, M2, and M3 are synthesized by LbL-assembly of 1, 2, and 4 bilayers of pDAC/PAA, respectively.

The surface charge of the PEs and the fabricated membranes was examined by measuring the surface zeta potential over different pH ranges, and the results are presented in Fig. 3a,b. As expected, over the tested pH range, the inherent surface charges of pDAC and PAA are positive and negative, respectively (Fig. 3a). The positive surface charge of pDAC originates from existing primary amine groups in its structure. Carboxylic (R–COO^−^) functional group in PAA macromolecule is responsible for the development of a negative surface charge^41, 42^.Based on the equilibrium dissociation reactions of amine and carboxylic functional groups in water, the surface charge is related to the degree of ionization and, consequently, the pH of the solution^43^. Hence, at higher pH values, pDAC becomes less positive while PAA becomes more negative.

Figure 3b illustrates the surface zeta potential over the pH range of 3–9 for the pristine PAI membrane and the LbL-assembled with different numbers of pDAC and PAA layers. The PAI pristine membrane has a negative surface charge over the entire pH range. The PAI membrane has an isoelectric point of 3, implying that at pH values less than 3, the protonation of amine groups switches the amphoteric surface zeta potential to positive values. The inherent negative charge of the pristine PAI membrane is attributed to the deprotonation of existing carboxyl groups^24, 44^. By coating the pristine membrane with one layer of pDAC, the surface charge of the membrane was reversed to a strongly positive charge due to the presence of the primary amine groups on the membrane surface, indicating the successful deposition of the first layer^45^. However, as the PAI membrane is modified with pDAC/PAA bilayers ending with a PAA layer, the outermost surface of the membranes has negative charges, originating from carboxylic groups of PAA. The zeta potential reversal, observed with deposition of bilayers, demonstrates the successful formation of bilayers. In all membranes, the zeta potential of all membranes became more negative with increasing pH. Childress et al.^46^ investigated the effect of pH on zeta potential for thin-film polyamide membranes at various concentrations of NaCl, Na_2_SO_4_, and CaCl_2_ solutions. At pH values more than the isoelectric point, the acid groups present in the membrane surface disassociates and the surface becomes more negatively charged. When the membrane is coated with a substance with abundant acidic functional groups such as carboxylic (–COOH) and hydroxyl groups (–OH), the membrane surface becomes rich in ionizable functional groups. With increasing pH, these groups are deprotonated to –COO^−^ and –O^−^ negative groups and increase the negative charge concentration over the membrane surface^47^.

Taking a closer look at Fig. 3b, it is evident that at higher pH values (pH > 7), deprotonation of carboxyl and hydroxyl functionals made different membrane surfaces very negative, which are very close to each other. As a result, electrostatic repulsion forces between all membranes’ surfaces and BFW would be similar. It means that zeta potential may not play a crucial role in the antifouling performances of different membranes.

### Evaluation of the chemical composition of the membranes

**Figure 4:**
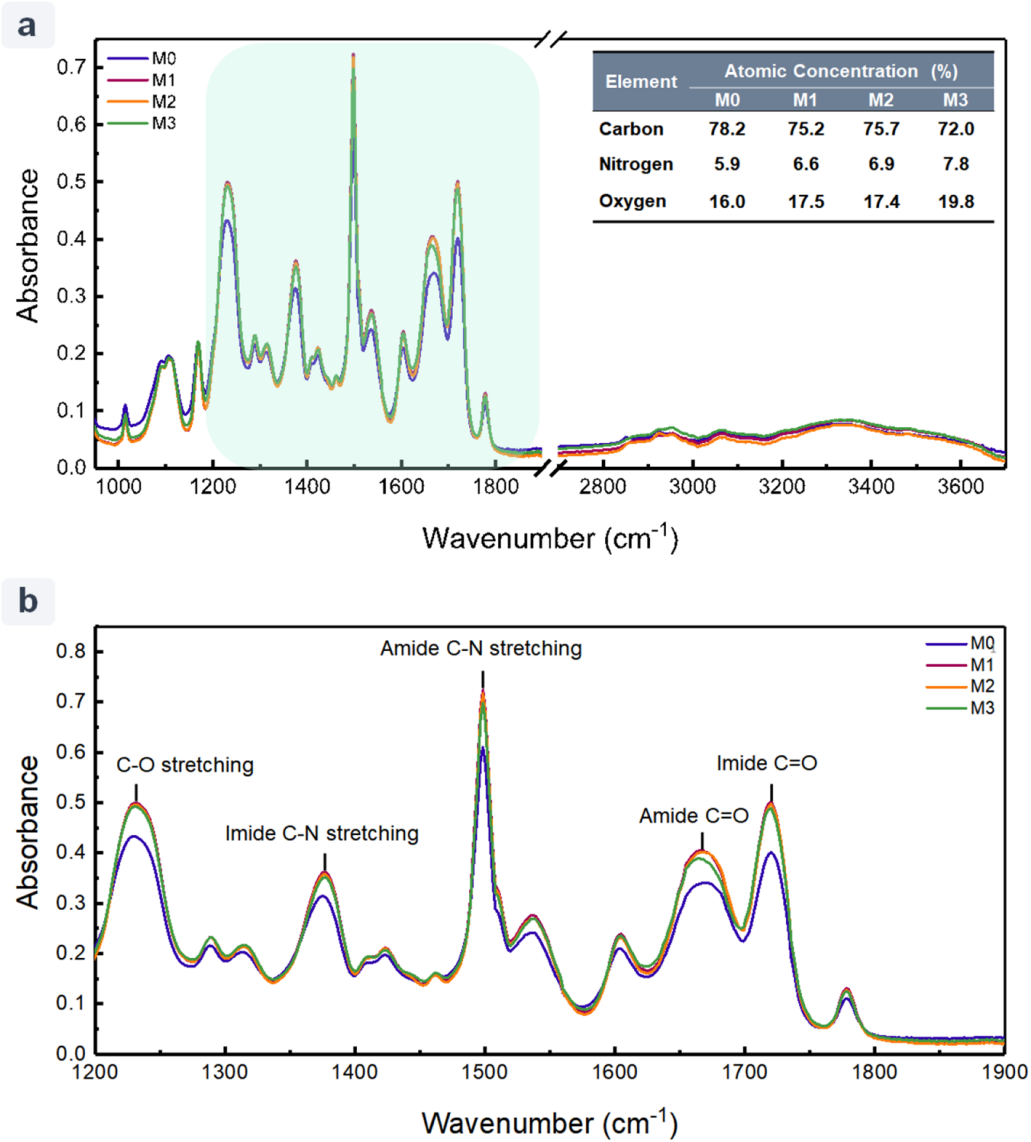
(**a**) ATIR-FTIR spectra and EDX data of the PAI support (M0) and the synthesized LbL-assembled membranes (M1, M2, and M3). (**b**) The magnified ATIR-FTIR spectra of the corresponding membranes. The modified membranes M1, M2, and M3 are synthesized by LbL-assembly of 1, 2, and 4 bilayers of pDAC/PAA, respectively.

EDX and ATR-FTIR spectroscopic techniques are employed to identify the elemental composition and the corresponding functional groups of the membrane surfaces. The EDX data of the fabricated membranes are presented in the inset tables of Fig. 4. For all the membranes, carbon, nitrogen, and oxygen are detected in EDX chemical elemental analysis. As can be seen, the intensity of the carbon peak is the highest in the pristine M0 (78.2%) and decreases as the number of bilayers increases from M2 (75.2%) to M4 (72.0%). Accordingly, the intensity of nitrogen and oxygen increases from M0 (N: 5.9% and O: 16.0%) to M4 (N: 7.8% and O: 19.8%), which can be attributed to the addition of amine (–NH_2_) and carboxyl acid (–COOH) functional groups of the PEs on the membrane surface.

Figure 4a represents the ATR-FTIR spectra of the pristine and the LbL-assembled membranes. Due to the high penetration depth of the IR beam, the ATR-FTIR spectra illustrate the peaks of the coated layers as well as the PAI substrate^48^. The PEs (pDAC and PAA) and the PAI substrate have similar functional groups; therefore, no new peaks are observed after surface modification. All spectra showed characteristic peaks at 1778 cm^−1^ (asymmetrical C=O stretching), 1720 cm^−1^ (symmetrical C=O stretching), 1378 cm^−1^ (C–N stretching), and amide peaks at 1670 cm^−1^ (C=O stretching) and 1500 cm^−1^ (C–N stretching). The magnified spectra, presented in Fig. 4b, clearly show that the intensity of these peaks enhanced as the number of bilayers increased from 1 to 4, indicating the successful LbL-coating of the PAI substrate^38^.

### Evaluation of roughness and surface wettability of the membranes

**Figure 5:**
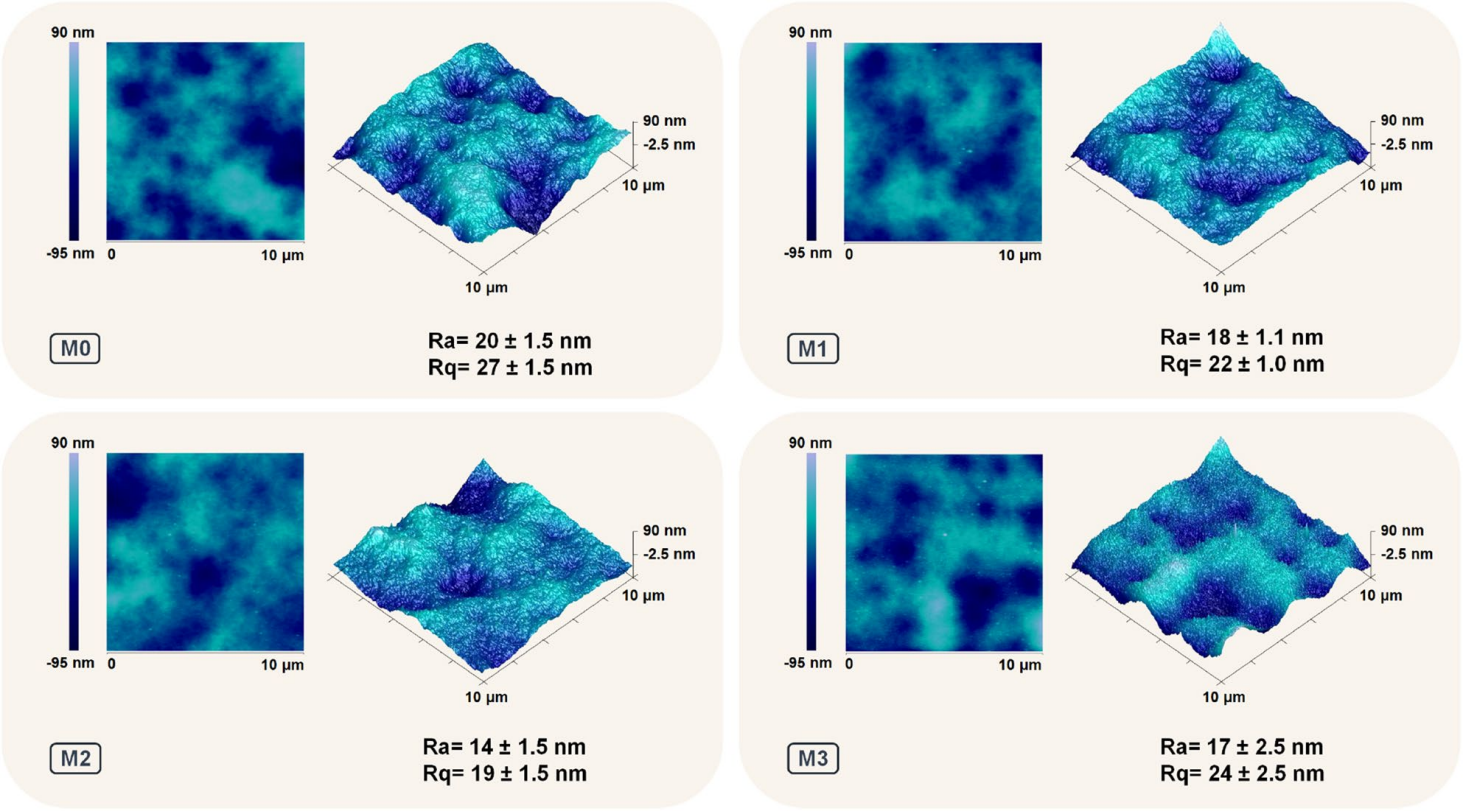
AFM surface topography images and surface roughness data of the pristine (M0), and modified M1, M2, and M3 membranes. The modified membranes M1, M2, and M3 are synthesized by LbL-assembly of 1, 2, and 4 bilayers of pDAC/PAA, respectively.

**Figure 6:**
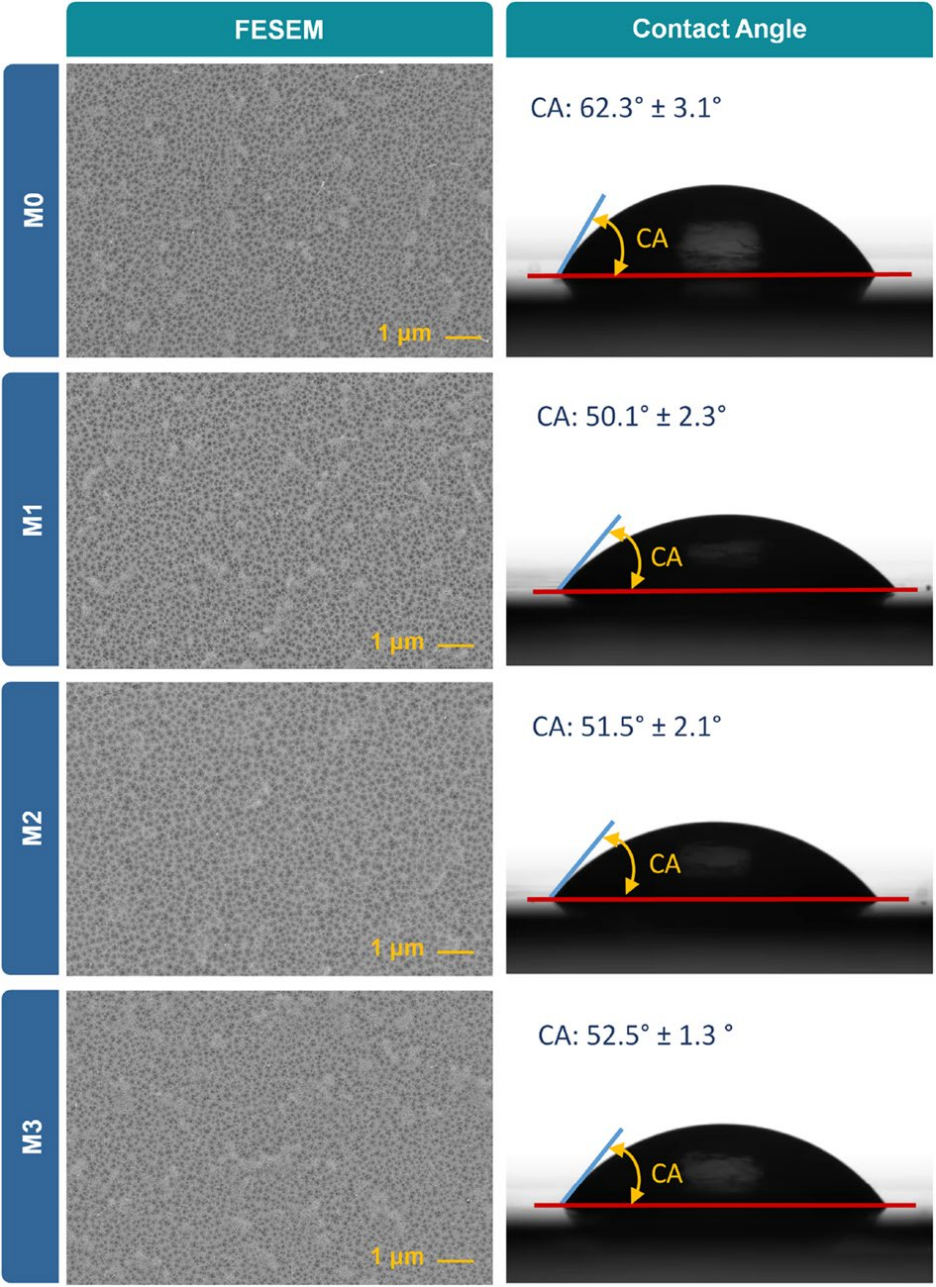
The top surface FESEM images, EDX data, and average water contact angles of the pristine PAI membrane (M0), and LbL-assembled membranes M1, M2, M3. The modified membranes M1, M2, and M3 are synthesized by LbL-assembly of 1, 2, and 4 bilayers of pDAC/PAA, respectively.

The surface roughness and morphological study of the synthesized membranes were investigated by AFM and FESEM, respectively. The arithmetic average surface roughness (R_a_) and the root mean square average surface roughness (R_q_) of the synthesized membranes are estimated using the AFM results and are presented in the inset of Fig. 5. The LbL-assembled membranes exhibit slightly lower roughness parameters compared to the pristine M0, indicating their smoother surfaces. The AFM 3D surface topographies in Fig. 5 show a more illustrative comparison of the membrane surface roughness. The top surface FESEM images of the fabricated membranes (Fig. 6) shows that PEs covered the surface of the pristine PAI membrane.

The surface wettability of the fabricated membranes is investigated by evaluating the contact angle of a water droplet on the membrane surface. Figure 6 presents the average water contact angle (WCA) of the fabricated membranes. The contact angle values of the LbL-coated membranes decreased by almost 10° compared to the pristine M0 membrane. The chemical composition and surface topography of membranes play an important role in surface wettability^49, 50^. Generally, the presence of hydrophilic functional groups on the surface of membranes can reduce WCA and increase surface wettability^51–53^. It has also been reported that the surface wettability of hydrophilic surfaces (WCA less than 90°) increases by increasing the surface roughness^51–53^. Hence, one may conclude that the wettability of the LbL-modified membranes should decrease as their roughness decreased slightly (Fig. 5). However, as evidenced by the ATR-FTIR results, the hydrophilicity of the LbL-modified membranes enhanced due to the increased intensity of the hydrophilic functional groups on the surface of these membranes. Therefore, the decrease of the WCA can be mainly attributed to the alteration of the chemical composition and improved surface hydrophilicity of the LbL-coated membranes.

### Evaluation of the permeation performance of the membranes

**Figure 7:**
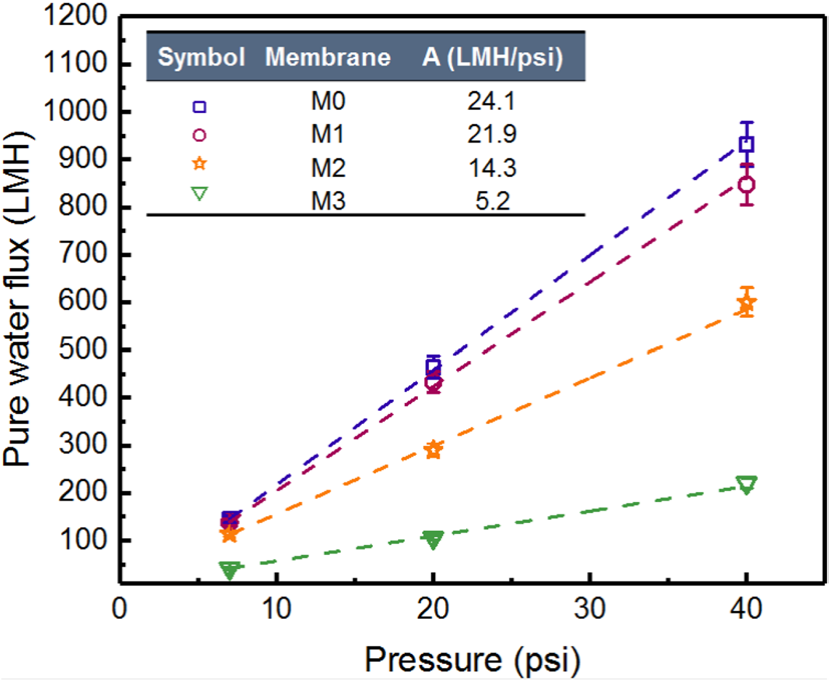
Pure water flux vs. TMP and hydrodynamic permeability of the synthesized membranes. The modified membranes M1, M2, and M3 are synthesized by LbL-assembly of 1, 2, and 4 bilayers of pDAC/PAA, respectively.

Figure 7 shows the pure water flux versus TMP of the synthesized membranes. The hydrodynamic permeability (A) of the membranes is presented in the table inside Fig. 7. By increasing the number of bilayers, hydrodynamic permeability of the fabricated membranes decreased from 24.1 LMH/psi for M0 to 21.9 LMH/psi, 14.3 LMH/psi, and 5.2 LMH/psi for M1, M2, and M3, respectively, which indicates the formation of a thicker LbL-film with higher mass transfer resistance at the surface of the pristine membrane. This observation indicates that the LbL-assembly of PAA and pDAC effectively blocked some open pores on the PAI support and led to the formation of tighter UF membranes with a narrower pore size structure.

### Evaluation of the separation performance and antifouling properties of the membranes

**Figure 8:**
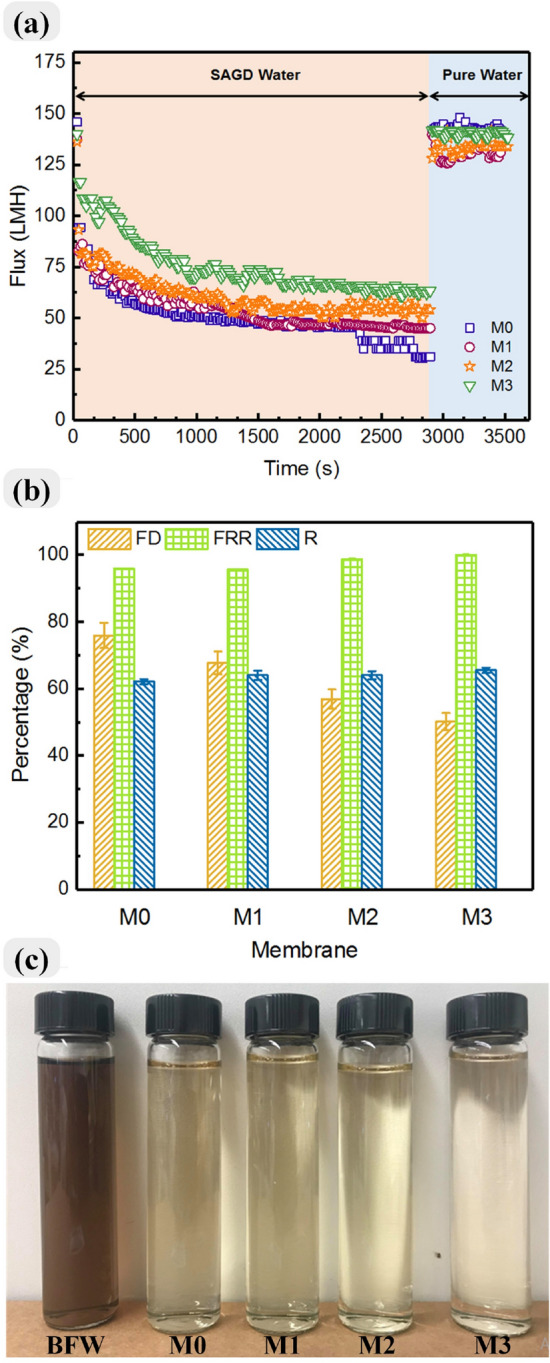
(**a**) Permeation flux, (**b**) antifouling characteristics of the pristine PAI membrane and the LbL-modified membranes, and (c) pictures of BFW and treated water by M0-M3 membranes. The modified membranes M1, M2, and M3 are synthesized by LbL-assembly of 1, 2, and 4 bilayers of pDAC/PAA, respectively.

The effect of the number of bilayers on the antifouling propensities and permselectivity of the LbL-modified membranes was examined by filtration of BFW. Figure 8 presents the antifouling characteristics of the pristine and LbL-coated membranes. A sharp flux decline is observed at the onset of the filtration tests, which is followed by a gradual flux decline (Fig. 8a). The initial permeate flux decline was continued even in the later stages of filtration for the pristine PAI membrane. In contrast, for the modified membranes, the permeate flux decline reached a steady condition. The initial sharp decline can be attributed to the partial pore blockage with the foulants, primarily organic matter^38^. The BFW mainly consists of high concentrations of organic matter and dissolved inorganic materials^54, 55^. Organic matter, such as aliphatic and aromatic hydrocarbons and humic substances, containing hydrophobic constituents in their structures, is dispersed in water^54, 55^. According to the SAGD BFW component specification presented in Table 1, the concentration of organic matter (500 mg L^−1^) is higher than divalent ions (30 mg L^−1^) in the BFW. Besides, due to the small particle diameter, these divalent ions (Ca^2+^ and Mg^2+^) will mostly pass through a microfiltration or UF membrane. Hence, organic fouling is expected to be the dominant fouling mechanism in this study^56^. These NOMS can result in an irreversible attachment to the surface of the membrane via hydrophobic-hydrophobic interactions^48^. In the later stage of filtration, the accumulation of foulants on the membrane surface leads to the cake layer formation due to the foulant-membrane or foulant-foulant interaction^56, 57^.

The flux decline ratio (FD) and the flux recovery ratio (FRR) of the synthesized membranes are presented in Fig. 8b. The unmodified M0 membrane exhibited the largest FD of 75.9% compared to the LbL-coated membranes. Increasing the number of bilayers lowers the FD to 50.2% for the M3 membrane. The relatively high FD of all synthesized membranes is attributed to the high concentrations of organic matter, dissolved and suspended solids in SAGD BFW^1^. However, the less FD of M3 than M0 can be attributed to its superior hydrophilicity, slightly smoother surface (Figs. 5 and 6), and tighter surface morphology. A hydrophilic membrane generally repels hydrophobic foulants more effectively as higher hydrophilicity favors interaction between the membrane surface and water over the interaction between membrane and hydrophobic foulants^58^. The foulants can also be physically entrapped in the valleys of a rough surface, and thus, a membrane with a smoother surface is less prone to fouling^2^. Finally, the tighter structure of the LbL-assembled membranes compared to the pristine PAI membrane can minimize the irreversible fouling due to the pore blockage^15^. FRR is another parameter that is widely used for the evaluation of fouling resistance of membranes. All membranes showed significantly high FRR, ranging from 95.9% for the unmodified M0 membrane to 100% for the 4-bilayer-coated M3 membrane. The low FD and the high FRR of the modified membranes demonstrate the feasibility of these membranes for oil sands produced water treatment and indicate that the foulants can be easily removed during washing and chemical cleaning processes^15^.

Figure 8b presents the performance of the fabricated membranes for the separation of the organic matter from SAGD BFW. The removal efficiency of the modified membranes slightly increased from 62.1% for the pristine M0 membrane to 65.5% for the M3 membrane, which can be due to the denser structure of the LbL-membranes. The reduction in the concentration of organic matter after membrane treatment can be visually observed in Fig. 8c. It is worth mentioning that the current water treatment schemes in the SAGD process, including warm lime softener and cation exchange resins, do not provide any treatment for organic matter^3^. The total dissolved solids (TDS) concentration even increases due to the use of weak acid cation exchanger for hardness removal. The high concentration of organic matter and TDS in BFW causes fouling of boiler tubes, thereby increasing the operating costs of the SAGD process^5^. Due to the low quality of BFW in current SAGD processes, a particular type of steam generator, called the once-through steam generator (OTSG), is used that can tolerate high amounts of organic matter and TDS^39^. However, OTSGs produce low-quality steam (~ 80%), which results in less oil production and a large volume of boiler blowdown water for disposal, adding to economic and environmental concerns for resource extraction in Alberta, Canada. The developed antifouling membranes in the present work address some of these concerns by removing up to 65% of organic matter from BFW.

## Conclusion

In this study, we employed the LbL assembly technique to enhance the antifouling properties of PAI membranes for the treatment of SAGD BFW. PAA and pDAC were used as polyelectrolytes and the influence of the number of the coating bilayers on the permeation performance and antifouling propensities of the fabricated membranes was studied. The modified membrane, coated with four bilayers of pDAC/PAA with 0.01 wt% concentration of the respective solutions, exhibited the highest surface hydrophilicity (water contact angle of 52° ± 2°), as well as the highest fouling resistance during filtration of SAGD BFW (FD of 50.2%, FRR of 100%). In contrast, the unmodified PAI membrane demonstrated the lowest fouling resistance (FD of 75.9%, FRR of 97.8%, and water contact angle of 62° ± 3°) under similar test conditions. Overall, the fabricated membranes can be employed for the pretreatment of SAGD produced water before delivering it into nanofiltration or reverse osmosis units. The pretreatment of the feed solution can significantly improve the performance of the water treatment process. Moreover, although the current SAGD produced water treatment process, including ion exchange resins and warm lime softening, can separate ~  90% of the divalent ions and silica, but it fails to remove the dissolved organic matter. Therefore, ~ 65% removal of organic matter can substantially reduce the risk of boiler failure and clogging the injection wells.

## Data Availability

All data generated or analyzed during this study are included in this published article.
